# Immunological properties of gold nanoparticles

**DOI:** 10.1039/c6sc03631g

**Published:** 2016-11-16

**Authors:** Lev A. Dykman, Nikolai G. Khlebtsov

**Affiliations:** a Institute of Biochemistry and Physiology of Plants and Microorganisms , Russian Academy of Sciences , 13 Prospekt Entuziastov , Saratov 410049 , Russia . Email: dykman_l@ibppm.ru ; Email: khlebtsov@ibppm.ru; b Saratov National Research State University , 83 Ulitsa Astrakhanskaya , Saratov 410012 , Russia

## Abstract

In the past decade, gold nanoparticles have attracted strong interest from the nanobiotechnological community owing to the significant progress made in robust and easy-to-make synthesis technologies, in surface functionalization, and in promising biomedical applications. These include bioimaging, gene diagnostics, analytical sensing, photothermal treatment of tumors, and targeted delivery of various biomolecular and chemical cargos. For the last-named application, gold nanoparticles should be properly fabricated to deliver the cargo into the targeted cells through effective endocytosis. In this review, we discuss recent progress in understanding the selective penetration of gold nanoparticles into immune cells. The interaction of gold nanoparticles with immune cell receptors is discussed. As distinct from other published reviews, we present a summary of the immunological properties of gold nanoparticles. This review also summarizes what is known about the application of gold nanoparticles as an antigen carrier and adjuvant in immunization for the preparation of antibodies *in vivo*. For each of the above topics, the basic principles, recent advances, and current challenges are discussed. Thus, this review presents a detailed analysis of data on interaction of gold nanoparticles with immune cells. Emphasis is placed on the systematization of data over production of antibodies by using gold nanoparticles and adjuvant properties of gold nanoparticles. Specifically, we start our discussion with current data on interaction of various gold nanoparticles with immune cells. The next section describes existing technologies to improve production of antibodies *in vivo* by using gold nanoparticles conjugated with specific ligands. Finally, we describe what is known about adjuvant properties of bare gold or functionalized nanoparticles. In the Conclusion section, we present a short summary of reported data and some challenges and perspectives.

## Abstract 2

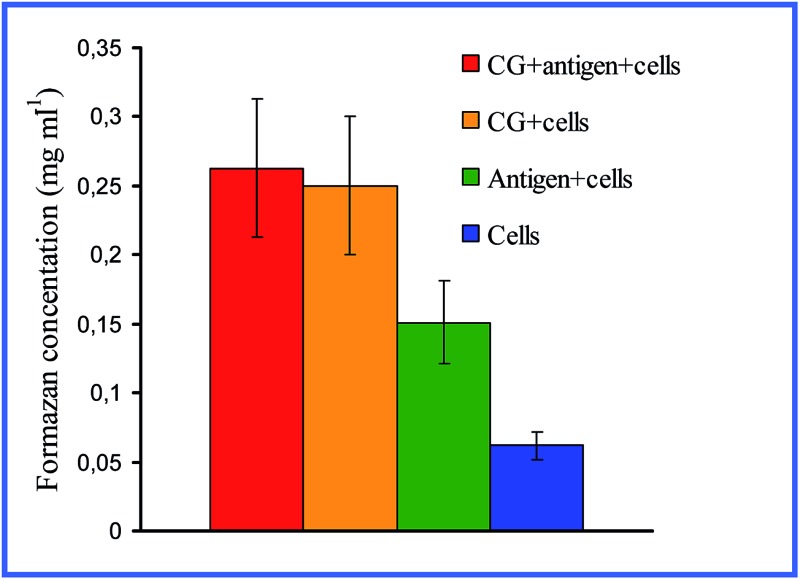
This review summarizes what is known about the application of gold nanoparticles as an antigen carrier and adjuvant in immunization for the preparation of antibodies in vivo and evaluating their potential for the development of effective vaccines.

## 1. Introduction

Gold nanoparticles (GNPs) have attracted significant interest as a novel platform in nanobiotechnology and biomedicine because of their convenient surface bioconjugation with molecular probes^1^ and their remarkable optical^2^ and immunological^3^ properties. Recently published examples include applications of GNPs to genomics, biosensorics, immunoassays, clinical chemistry, detection and control of microorganisms, cancer cell photothermolysis, targeted delivery of drugs or other substances, and optical imaging and monitoring of biological cells and tissues.^4–6^ Noteworthy is the fact that GNPs are being increasingly administered to animals and humans parenterally. In particular, they serve as carriers for the delivery of drugs, genetic materials, and antigens. “Colloidal metallic gold is not bio-inert”—such is the name Brown *et al.* ^7^ gave to their article so as to stress the importance of nanometer size in biological effects, even for such a seemingly inert material as gold.

It is natural to suppose that the first cells that GNPs encounter on their way in the mammalian organism are those of the immune system, in particular its phagocytic link (neutrophils, monocytes, macrophages, dendritic cells and mast cells). Indeed, as early as in the first attempts to investigate colloidal gold biodistribution, which were performed in the 1960s–80s on rabbits,^8^ mice,^9^ and rats^10,11^ it was found that after parenteral administration, colloidal gold particles are captured by liver cells, excreted through bile, and eliminated from the organism with feces. After injection, gold was identified mostly in Kupffer cells. Perhaps Scott *et al.* ^8^ were the first to note that the phagocytosis of GNPs is size dependent. Besides Hardonk *et al.*,^10^ the important role of Kupffer cells in the elimination of GNPs was established by Sadauskas *et al.*,^12^ who injected GNPs intravenously in mice. Electron microscopy showed that after injection, the GNPs accumulated in the macrophages of the liver (90%) and spleen (10%). The authors concluded that GNPs penetrate only phagocytes, primarily the Kupffer cells of the liver. In a subsequent study,^13^ Sadauskas *et al.* reported that GNPs get localized in lysosomes (endosomes) of Kupffer cells and can be retained there for up to six months. The influence of size, solubility and surface modification on the biocompatibility of GNPs and their use in biological applications is well known.^14,15^ However, the effects of nanoparticle properties on the immune system are still being explored.

In this review, we discuss the selective penetration of GNPs into immune cells and the interaction of GNPs with immune cell receptors. This review also summarizes what is known about the application of GNPs as an antigen carrier and adjuvant in immunization for the preparation of antibodies *in vivo*.

## 2. Interaction of gold nanoparticles with immune cells

The immune system cells constitute the first barrier to nanoparticle penetration of animal tissues and cells. Therefore, the study of GNP interactions with phagocytes, the mechanisms of intracellular uptake, and the responses of immune cells to GNPs is undoubtedly of major interest. Perhaps the first detailed consideration of these issues can be found in Shukla *et al.*,^16^ who, using three microscopic methods, examined the uptake of 3 nm GNPs into RAW264.7 macrophage cells. The conclusion from their study was that small GNPs enter macrophages through pinocytosis and get localized mostly in lysosomes and in the perinuclear space. On the whole, Shukla *et al.*'s data indicate that the GNPs are biocompatible, noncytotoxic and nonimmunogenic and that they suppress the production of reactive oxygen species and do not cause elaboration of the proinflammatory cytokines TNF-α and IL1-β (which contradicts the data of Yen *et al.* ^17^). In contrast to data by Shukla *et al.*,^16^ Yen *et al.* ^17^ noted that on the administration of GNPs, the number of macrophages decreases and their size increases, this being accompanied by elevated production of IL-1, IL-6 and TNF-α. We emphasize that the data of Shukla *et al.* ^16^ were obtained for very small (3 nm) particles. However, Lim *et al.*,^18^ using much larger (60 nm) hollow NSphs capped with dextran, and Zhang *et al.*,^19^ using 60 nm GNPs, achieved results similar to the findings of Shukla *et al.* ^16^ for the same cell culture. Sumbayev *et al.* ^20^ showed that citrate-stabilized GNPs specifically downregulate, in a size dependent manner, the cellular responses induced by IL-1β both *in vitro* and *in vivo*. In a recent study, Guevél *et al.* ^21^ demonstrated that 12 nm gold nanoparticles induce cell mediated responses accompanied by inflammatory natural killer (NK) cell stimulation, whereas 2 nm gold nanoparticles are more efficiently taken up without inducing dendritic cell maturation or lymphocyte proliferation. To summarize, the published data revealed strong effects of the GNP size and functionalization on production of proinflammatory cytokines.

With some inspiration from data on GNP uptake by macrophages, Choi *et al.* ^22^ even proposed a new method for the photothermal therapy of tumors that employs a “Trojan horse” in the form of monocytes and macrophages laden with phagocytosed GNSs. For these purposes, Dreaden *et al.* ^23^ suggested the use of GNPs conjugated with macrolide antibiotics, which can accumulate in tumor-specific macrophages and induce their cytotoxicity, causing tumor cells to die. Thus, particle size and structure in these studies were not critical to macrophage uptake.

The influence of colloidal gold on immunocompetent cells was examined *in vivo* also by Tian *et al.* ^24^ and by Lou *et al.* ^25^ In particular, injection of nonconjugated GNPs into mice enhanced the proliferation of lymphocytes and normal killers, as well as increasing the IL-2 production.

**Fig. 1:**
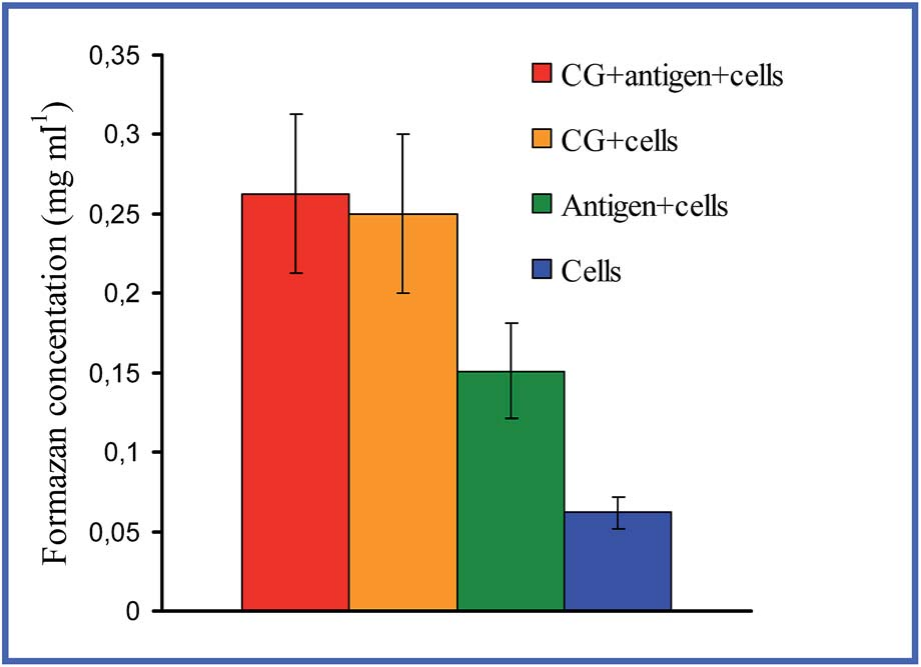
Changes in the concentration of reduced formazan depending on the cultivation conditions of antigen (AG) with peritoneal rat macrophages. Reproduced with permission from ref. 28, © 2009, Springer.

**Fig. 2:**
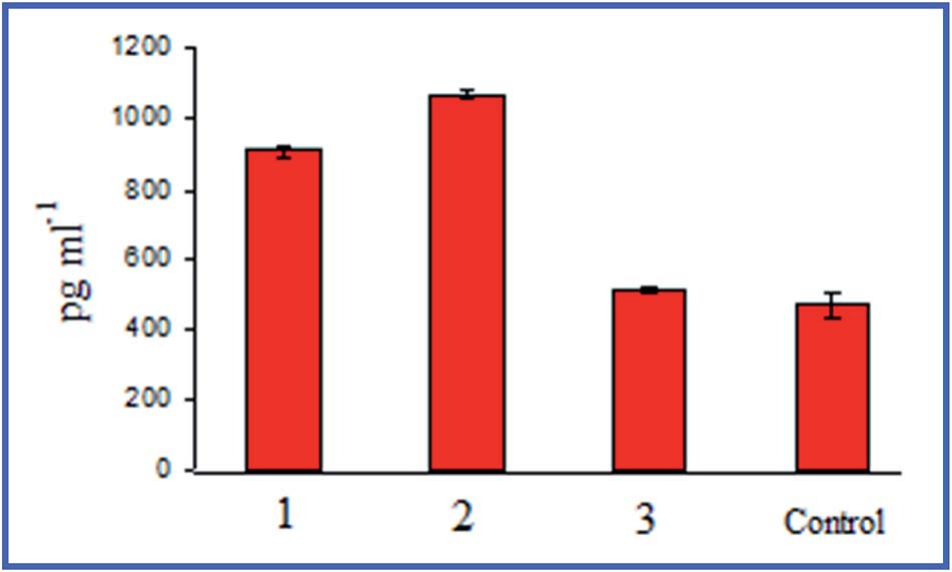
Changes in the serum IFN-γ concentrations in rats immunized with different antigens. 1 – immunization with native antigen; 2 – immunization with antigen conjugated with GNPs; 3 – immunization with GNPs. Reproduced with permission from ref. 29, © 2011, Springer.

Quite interesting data were acquired by Bastús *et al.* ^26,27^ with 10 nm nonconjugated GNPs. From their results, it follows that indeed, on entry into murine bone marrow macrophages, GNPs do not affect the production of proinflammatory cytokines. However, if the GNP surface is modified with the peptide AGIP (amyloid growth inhibitory peptide, LPFFD) or SAP [sweet arrow peptide, (VRLPPP)_3_], GNPs, on entry into the macrophages, involve the induction of NO synthase and proinflammatory cytokines such as TNF-α, IL-1β and IL-6. In addition, they inhibit macrophage proliferation. The recognition of GNP–peptide conjugates was made more effective through toll-like receptors 4 (TLR-4) on the surface of the macrophages. Yet, Staroverov *et al.* ^28,29^ demonstrated that both 15 nm nonconjugated GNPs and their conjugates with high- and low-molecular-weight antigens, on entry into rat peritoneal macrophages, enhance their respiratory activity and the activity of macrophage mitochondrial enzymes (Fig. 1). GNPs also have greatly increased the production of IL-1, IL-6 and IFN-γ (Fig. 2). Lee *et al.* ^30^ reported that the penetration of gold nanorods (GNRs) and SiO_2_-coated GNRs into macrophages induces the release of inflammatory mediators (cytokines, prostaglandins, *etc.*) and the activation of immune response genes. Thus, in addition to early observations by Shukla *et al.* ^16^ for bare GNPs, the published data^26–29^ indicate a significant role of surface coating in macrophage response after GNP uptake.

The activation of macrophages by GNPs, found by several authors,^26–28,30–34^ can serve as a basis for new vaccine adjuvants. As in the usual cellular uptake, immunoactivity depends strongly on the particle size: 5 nm particles conjugated with disaccharides performed far better than smaller, 2 nm ones.^35^

Yet another means of activating macrophages with GNPs was proposed by Wei *et al.* ^36^ For this purpose, they used 15 and 30 nm GNPs conjugated to cytosine–phosphate–guanosine (CpG) oligodeoxynucleotides. As is known, these oligonucleotides are demethylated sites of microbial DNA that can activate macrophage immune response by interacting with the TLR-9 receptors and subsequently triggering a cascade of immune response signals. The immunostimulating activity of synthetic oligonucleotides containing CpG motifs may be analogous to that of oligonucleotides from bacterial DNA.^37^ According to Wei *et al.*,^36^ GNP–CpG conjugates were effective in enhancing nanoparticle internalization in RAW264.7 macrophages, and they greatly increased the secretion of proinflammatory cytokines such as TNF-α and IL-6 (15 nm conjugates did so to a greater degree than 30 nm ones did). The immunostimulatory effect of GNP–CpG was much greater than that of native CpG at the same concentrations.

A recent study^38^ examined the influence of the size of PEGylated GNPs on the activation of the TLR-9 receptors of RAW264.7 murine macrophages by CpG oligonucleotides. GNPs with diameters of 4, 11, 19, 35 and 45 nm inhibited CpG-induced elaboration of TNF-α and IL-6 and the activity of the TLR-9 receptors. This effect was markedly size dependent, with a peak for 4 nm GNPs, which penetrated the cells most intensively.

Massich *et al.* ^39^ reported on the immune response of macrophages after the phagocytosis of GNPs functionalized with polyvalent oligonucleotides. The effectiveness of uptake and the level of interferon production were found to depend on the density of DNA molecules on the GNP surface. Kim *et al.* ^40^ showed that the uptake effectiveness of oligonucleotide-functionalized GNPs differs for cells isolated from peripheral blood (mononuclear cells) and those introduced into a 293T culture. In addition, only in the first type of cell did the uptake of GNP conjugates activate the expression of immune response genes.

**Fig. 3:**
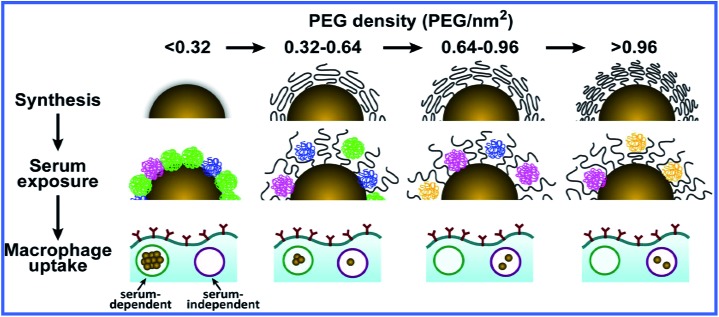
Scheme for the influence of the PEG coating density on the adsorption of serum proteins to GNPs and their subsequent uptake by macrophages. Reproduced with permission from ref. 41, © 2012, American Chemical Society.

A recent article by Walkey *et al.* ^41^ described a thorough study of the effect of coating GNPs with serum proteins and PEG on macrophage uptake. The authors studied the adsorption of 70 blood serum proteins to PEG-coated GNPs with different densities of PEG coating. Increasing the PEG coating density reduced serum protein adsorption and changed the composition of the adsorbed protein layer. Particle size also affected serum protein adsorption through a change in the steric interactions between the PEG molecules. Both the density of PEG molecules on the GNP surface and the size of GNPs determined the mechanism and effectiveness of macrophage uptake, possibly because the composition of the adsorbed blood serum proteins and their availability to cells were regulated. If the density of PEG coating was lower than ∼0.16 PEG molecules per nm^2^, the macrophage uptake of GNPs depended on the presence of adsorbed proteins (serum-dependent uptake). If the density was higher than ∼0.64 PEG molecules per nm^2^, serum-independent uptake was seen (Fig. 3).

Serum-dependent uptake was more effective than serum-independent uptake, apparently because of the difference in the energy of the GNP–cell interaction. Interestingly, serum-independent uptake was more effective for large GNPs (90 nm) whereas serum-dependent uptake was maximal for 50 nm GNPs.

It should be noted that immediately on contact of GNPs with blood, lymph, gastric juice, or any other biological liquid *in vivo* the interaction between GNPs and solvable proteins and other biomolecules results in the formation of a protein “corona”.^42,43^ Similarly to the concept of functionalized GNPs, the concept of a GNP–protein corona is important in tuning the surface physicochemical properties of GNPs, such as charge, hydrodynamic size and colloidal stability. In fact, it is the GNP–protein corona that forms the first nano–bio interface and determines the first interactions of GNPs with/or within living cells. This is because the GNP–protein corona is a dynamic biopolymer layer that can strongly affect cellular uptake owing to modification of the particle properties (the overall size, charge, *etc.*). Although as much as 69 plasma proteins can bind to the GNP surface,^44,45^ only some of them, such as albumin, apolipoprotein, immunoglobulin, complement and fibrinogen, are the most abundantly bound proteins forming the GNP–protein corona. After intravenous injection, the coating of GNPs by these proteins largely determines the particles' fate in the body—biodistribution over organs, tissues and cells, the efficiency of cellular uptake and clearance, immunological properties, and so on.^46,47^

Ma *et al.* ^48^ showed that GNPs attenuate LPS-induced NO production through the inhibition of nuclear factor-κB and IFN-β/STAT1 pathways in RAW264.7 cells. In contrast, Liu *et al.* ^49^ demonstrated that PEGylated GNPs were internalized more quickly by lipopolysaccharide-activated RAW264.7 cells than by unstimulated cells, reaching saturation within 24 h. The PEGylated GNPs enhanced LPS-induced production of NO and IL-6 and inducible nitric oxide synthase expression in RAW264.7 cells, partly by activating p38 mitogen-activated protein kinases and NF-κB pathways. Goldstein *et al.* ^50^ showed that GNPs and their plasmonic excitation could activate the Nrf2-Keap1 pathway in macrophages.

García *et al.* ^51^ studied the cellular uptake of GNPs with or without exposure of cells to latrunculin A, a phagocytosis inhibitor. The results indicate a size dependence of the internalization mechanisms for macrophage (THP-1) cells. The internalization of larger GNPs (15 and 35 nm) was blocked in the presence of latrunculin A, although they could attach to the cell membrane. Smaller GNPs (5 nm), though, were not blocked by actin-dependent processes.

Of considerable interest are studies on the uptake of GNPs not only by macrophages but also by other cells of the immune system, in particular dendritic cells. In the past decade, dendritic cells have attracted increased interest owing to the ease of their isolation from peripheral blood monocytes and to their ability to effectively present antigens to T cells. By now, a great deal of work has been done on the modulation of immune response in patients with chronic infections and oncological diseases by using antigen-primed dendritic cells.^52^ GNPs have been named, among other carriers, for application in antigen delivery to dendritic cells. For example, Cheung *et al.* ^53^ described the use of 15 nm GNPs for presenting a peptide antigen associated with Epstein–Barr virus to dendritic cells. According to their TEM data, peptide-functionalized GNPs penetrated the dendritic cell cytoplasm but were not found in the nuclei. The uptake of GNPs by dendritic cells resulted in an increased content of γ-interferon, the presentation by major histocompatibility complex I (MHC-I) of the antigen to CD4+ T cells, and, correspondingly, activation of an epitope-specific immune response by cytotoxic T cells.

Cruz *et al.* ^54^ addressed dendritic cell uptake of and immune response activation by 13 nm GNPs conjugated to prostate cancer peptide antigens. By TEM, LCM and flow cytometry, GNPs functionalized with the peptides and with Fc fragments of IgG were shown to interact with the Fcγ receptors of dendritic cells and were localized, upon uptake, in the cytoplasm in a diffuse way. Internalization of antigen-conjugated GNPs in dendritic cells brought about an increase in the immune response, as compared with the effect obtained from the use of the native antigen, which was manifested as enhanced lymphocyte proliferation. Such an approach, in the authors' opinion, opens up the way to the creation of an effective system for the development of antitumor and other vaccines.

Villiers *et al.* ^55^ reported the effect of 10 nm non-antigen-functionalized GNPs on the immune functions of dendritic cells. From their findings, the GNPs that had entered cell endosomes were not cytotoxic and had no effect on the production of the proinflammatory cytokine IL-6. However, they did promote the secretion of interleukin IL-12p70, which is directly involved in the activation of T cells and, thus, in the regulation of an antigen-specific immune response. The authors also noted the development of long dendrites and an increase in the cell-surface amount of MHC-II molecules, which present antigens to T lymphocytes. Thus, even nonfunctionalized GNPs are immunostimulatory to both dendritic cells and macrophages.^17^

Ye *et al.* ^56^ used TEM and flow fluorocytometry to quantify the uptake of GNRs by dendritic cells and the particle effect on their functions. Compared to spherical GNPs, GNRs entered dendritic cells more effectively and induced higher expression of CD86 immunocostimulatory molecules, which are characteristic of dendritic cells.

Lin *et al.* ^57^ reported that GNPs in complexes with peptides derived from tumor-associated antigens are taken up effectively by dendritic cells. Moreover, dendritic cells take up GNPs with minimal toxicity and can process the vaccine peptides on the particles to stimulate cytotoxic T lymphocytes. A high peptide density on the GNP surface can stimulate cytotoxic T lymphocytes better than can free peptides. Thus, GNPs have great potential as carriers for various vaccine types.

GNP-mediated response of dendritic cells depends on the physicochemical properties of the GNP surface. For example, Fytianos *et al.* ^58^ clearly indicated that the chemical composition and surface charge of GNPs modulate uptake by dendritic cells and cytokine release. Further, *in vivo* GNP effects are dose-dependent. In particular, Małaczewska^59,60^ demonstrated that mice, after being orally administered with GNPs, showed an increased activity of phagocytes and some changes in the lymphocyte phenotypes, *i.e.*, an increased percentage of B and CD4+/CD8+ double positive T cells. The lowest dose had a pro-inflammatory or immunostimulating effect, enhancing the synthesis of proinflammatory cytokines (IL-1β, IL-2, IL-6, TNF-α). The effect of the highest dose can be considered proinflammatory or immunotoxic, because the stimulated cytokine synthesis was accompanied by a drastic decline in the proliferative activity of lymphocytes.

To estimate the functional impact of GNPs on B lymphocytes, Sharma *et al.* ^61^ treated a murine B lymphocyte cell line (CH12.LX) with 10 nm citrate-stabilized GNPs. This treatment activated an NF-κB-regulated luciferase reporter, and this activation correlated with the altered B lymphocyte function (*i.e.*, with increased antibody expression). According to TEM images, GNPs could penetrate the cellular membrane and, therefore, could interact with the intracellular components of the NF-κB signaling pathway.

*In vitro*, *ex vivo* and *in vivo* evidence suggests that GNPs activate B cells and enhance IgG secretion.^62^ GNP treatment upregulates blimp1, downregulates pax5, and enhances downstream IgG secretion. This enhancement is size and time dependent. GNPs ranging from 2 to 12 nm had the maximum stimulatory activity for the production of antibody.

Moreover, GNPs augmented lymphocyte proliferation in response to phytohemagglutinin, and this effect was greater for as-synthesized than for capped gold nanoparticles. Release of IL-10 and IFN-γ from lymphocytes was increased and the effect was again more marked for as-synthesized GNPs than it was for capped GNPs.^63^

Bartneck *et al.* ^65,66^ reported the interaction of variously shaped and sized particles GNPs with human neutrophil granulocytes, monocytes and macrophages. On the basis of their study, the mechanism of nanoparticle trapping can be classified as macropinocytosis rather than phagocytosis. Particle shape was found to affect strongly the particle trapping by cells of the immune system; specifically, CTAB-coated GNRs (50 × 15 nm) could be trapped faster than CTAB-coated 15 and 50 nm gold nanospheres. Replacing CTAB by poly(ethylene oxide) greatly reduced uptake effectiveness for both types of GNPs. Nanoparticle uptake by the immune cells was accompanied by an activation of the genes of proinflammatory cytokines and by a corresponding change in the cell phenotype. A characteristic fact is that the “professionally” phagocytic cells took up GNPs two orders of magnitude more effectively than did, *e.g.*, HeLa cells. In addition, Bartneck *et al.* revealed an alternative elimination mechanism whereby GNPs can be cleared from peripheral blood *via* an extracellular network (“trap”) produced by neutrophil granulocytes.

The same group presented data^67^ on the uptake of GNPs into various cells of the reticuloendothelial system: monocytes, macrophages, immature and mature dendritic cells and endothelial cells. The greatest uptake ability was demonstrated by macrophages, endothelial cells and immature dendritic cells. Positively charged GNPs penetrated into cells of the reticuloendothelial system more effectively. Moreover, GNPs intensified the induction of several cytokines, including γ-interferon, IL-8 (both in dendritic cells and in macrophages), IL-1β and IL-6 (only in dendritic cells). Interestingly, in mature dendritic cells, GNPs accumulate in the MHC-II compartment and, consequently, may affect antigen processing.

**Fig. 4:**
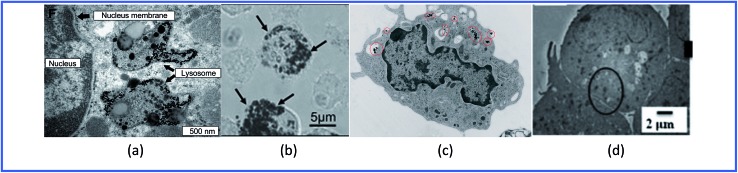
TEM images of (a) spleen macrophages, (b) dendritic cells, (c) monocytes and (d) lymphocytes treated with GNPs. Reproduced with permission from ref. 64, © 2009, Elsevier; ref. 55, © 2010, Springer; ref. 65, © 2010, American Chemical Society; and ref. 61, © 2013, The Royal Society of Chemistry.

Thus, GNPs can penetrate into various immune cells (Fig. 4) and activate the production of proinflammatory cytokines (Table 1).

**Table 1:** Effect of GNPs on the functions of various immune cells

Macrophages	Dendritic cells	Lymphocytes
Induction of cytokines (IL-1β, IL-6, IL-8, IL-10, TNF-α) and prostaglandins Stimulation of CD8+ and CD4+ T cells Activation of immune response genes Inhibition of macrophage proliferation, decreasing their amount and increasing their size Activation of Keap1/Nrf2 signaling pathway	Induction of IFN-γ, TNF-α, IL-1β, IL-6, IL-8, IL-12p70 cytokines Stimulation of CD8+ and CD4+ T cells Induction of CD86 costimulatory molecules Increasing the cell-surface amount of MHC-II Increasing the amount of dendritic cells Activation of antigen processing	Induction of IL-2 and IFN-γ cytokines Increasing proliferation of lymphocytes and NK cells Activation of NF-κB signaling pathway Regulation of blimp1/pax5 signaling pathway Enhance antibody secretion in B cells

Phagocytic cells of the immune system have a multitude of various receptors on their surface, through which they bind and take up foreign material.^68,69^ The interactions with various types of receptors and, consequently, various types of GNP endocytosis depend in many ways on nanoparticle size and shape but especially on surface functionalization (including opsonization by proteins from the culture medium or blood plasma)^70^ and on the presence of mannose-containing polysaccharides on the GNP surface.^71^ Some researchers are inclined to believe that the key role in macrophage uptake of GNPs is played by scavenger receptors.^72,73^ These are mainly involved in the endocytosis of apoptotic cells. A characteristic peculiarity of their functioning, in contrast to the other macrophage receptors, is the absence of release of proinflammatory cytokines.

**Fig. 5:**
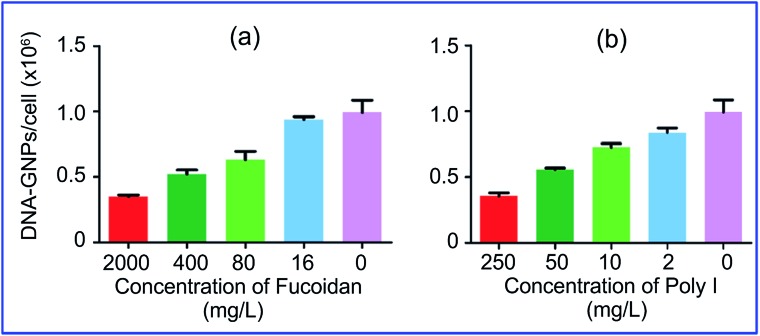
Cellular endocytosis of GNPs is mediated by scavenger receptors. Cell preincubation with fucoidan (a) and polyinosinic acid (b), which are agonists for these receptors, decreased the uptake by 60%. Reproduced with permission from ref. 74, © 2010, American Chemical Society.

More specifically, Patel *et al.* ^74^ demonstrated that the uptake of GNPs functionalized with polyvalent oligonucleotides by mammalian cells is effected through scavenger receptors. Cell preincubation with fucoidan and polyinosinic acid, which are agonists for these receptors, decreased the uptake by 60% (Fig. 5). However, bafilomycin A1 and methyl-β-cyclodextrin did not inhibit GNP uptake, because these pharmacological agents are known to inhibit other modes of cellular entry. Coating of GNP conjugates with serum proteins also reduced uptake effectiveness.

An in-depth study on the involvement of scavenger receptors in macrophage uptake of GNPs was published by França *et al.* ^75^ Their data show that macrophages take up opsonized GNPs through SR-mediated pathways (both 30 and 150 nm GNPs), as well as through clathrin- and caveolin-dependent pinocytosis (only 30 nm GNPs). Thus, the smaller 30 nm particles use a broader range of internalization routes, in contrast to the larger 150 nm GNPs. Noteworthy is the fact that as demonstrated by inhibition analysis, phagocytosis began with an interaction of GNPs with scavenger receptors and was not attended by induction of proinflammatory cytokines.

## 3. Production of antibodies by using gold nanoparticles

Since the 1920s, the immunological properties of colloidal metals (in particular, gold) have been attracting much research interest. This interest is mainly due to the physicochemical (nonspecific) theory of immunity proposed by J. Bordet, who postulated that immunogenicity, along with antigenic specificity, depends predominantly on the physicochemical properties of antigens, first of all on their colloidal state. L. A. Zilber made successful attempts to obtain agglutinating sera to colloidal gold^76^ (interestingly, a repeated attempt to prepare antisera to colloidal gold was performed almost 80 years later, in 2006).^77^ Yet, several authors have shown that the introduction of a complete antigen together with colloidal metals promotes the production of antibodies.^78^ Furthermore, some haptens may cause antibody production when adsorbed to colloidal particles.^79^ Numerous data on the influence of colloidal gold on nonspecific immune response are given in one of the best early reviews.^80^ In particular, it was noted that at 2 h after an intravenous injection of 5 mL of colloidal gold into rabbits, there was a sizable increase in total leucocytes in 1 mL of blood (from 10 000 to 19 800) against a slight decline in mononuclear cells (from 5200 to 4900) and a considerable increase in polynuclear cells (from 4700 to 14 900).^81^ On injection of other colloidal metals, no such phenomena were observed. Unfortunately, with advances in immunology and with denial of many postulates of Bordet's theory, interest in the immunological properties of colloids decreased. There is no doubt, though, that the data obtained on the enhancement of immune response to antigens adsorbed on colloidal particles were utilized for the development of various adjuvants.^82,83^

The size-dependent GNPs-induced changes (both increasing and decreasing) of the number of white blood cells have been reported in several recent publications.^84–86^

It is known that antibody biosynthesis is induced by substances possessing sufficiently developed structures (immunogenicity). The substances include proteins, polysaccharides, and some synthetic polymers. However, many biologically active substances (vitamins, hormones, antibiotics, narcotics, *etc.*) have relatively small molecular masses and, as a rule, do not elicit a pronounced immune response. In standard methods of antibody preparation *in vivo*, this limitation is overcome by chemically attaching such substances (haptens) to high-molecular-weight carriers (most often proteins), which makes it possible to obtain specific antisera. However, such antisera usually contain attendant antibodies to the carrier's antigenic structures.^87^

Let us take a brief look at two interrelated problems in current immunology that have attracted much research attention. These are the development of antibodies to nonimmunogenic low-molecular-weight compounds (haptens) and the creation of next-generation vaccines based on natural (microbial) or synthetic peptides.^88–93^ It is known that antibody biosynthesis is induced by substances possessing sufficiently developed structures (immunogenicity). These substances include proteins, polysaccharides, and some synthetic polymers.^94^ However, many biologically active substances (neurotransmitters, hormones, vitamins, antibiotics, *etc.*) have relatively small molecular masses. Low-molecular-weight antigens are in the category “weak antigens,” *i.e.*, they do not elicit a pronounced immune response.

Because haptens are weakly immunogenic, the choice of an optimal carrier (delivery system) providing a high immune response, in parallel with the obtainment of pure enough antibody preparations, is an important task when producing antibodies to low-molecular-weight compounds. Traditionally, this problem is solved by chemical attachment of a hapten to a protein matrix called a schlepper (from the German *schleppen* “to drag”), and by the use of adjuvants and intensive schemes of animal immunization with the obtained conjugate.^87,95^ Bovine serum albumin (BSA), ovalbumin, thyreoglobulin, hemocyanin and diphtheria or tetanus toxoids (in the case of synthetic peptides) are generally used as schleppers. However, this method yields antibodies to both the hapten and the immunodominant sites of the carrier. Note that when such a carrier is used, a pronounced immune response to weak antigens does not always develop. Besides, the subsequent purification and screening of the obtained antibodies are laborious and expensive, and their titre and affinity are often low. Most currently used adjuvants based on oil emulsions and on suspensions of inorganic substances are, as a rule, liable to phase separation, are often reactogenic, and their immunogenic properties vary with time. Many of these adjuvants cause local and systemic toxicity.^82^

In recent years, efforts have been made to develop “complex antigens”, *i.e.*, artificial molecular complexes formed from both necessary antigens and carriers or/and adjuvants. In particular, synthetic polyelectrolytes (poly-l-lysine, polyacrylic acid, polyvinylpyridine, sulfonated polystyrene, ficoll, *etc.*) were proposed for use as adjuvants.^96^ These polymer compounds are produced by chain-radical polymerization of the corresponding monomers. The simplicity of polyelectrolyte composition and synthesis, the possibility of obtainment of polymer chains with a wide range of molecular masses (*i.e.*, of various lengths), their solubility in water, and other properties (the capacity for conformational transitions, the formation of complexes with proteins, *etc.*) opened up possibilities for the use of polyelectrolytes in immunologic investigations. Such adjuvant carriers are capable of antigen deposition at the sites of injection, enhancement of antigen presentation to immunocompetent cells, and induction of production of necessary cytokines. However, the low immunogenicity of such complexes, due to their small epitope density, prompts researchers to look for new nontoxic and effective carriers additionally possessing adjuvant properties.

In this respect, of special interest are nanoscale corpuscular carriers: polymer nanoparticles [*e.g.*, those made of polymethylmethacrylate, polyalkylcyanoacrylate, polylactide-*co*-glycolide, poly(γ-glutamic acid), polystyrene, *etc.*]; liposomes, proteasomes and microcapsules; fullerenes; carbon nanotubes; graphene oxide; dendrimers; paramagnetic particles; silica nanoparticles; titanium dioxide nanoparticles; aluminum and aluminum oxide nanoparticles; cobalt oxide nanoparticles; silver nanoparticles; selenium nanoparticles, and others. When these are used, the forms of manifestation of immunogenicity of a given substance in the host's immune system vary. An antigen, once adsorbed or encapsulated by nanoparticles, may be used as an adjuvant for optimization of the immune response after vaccination.^97–102^

**Fig. 6:**
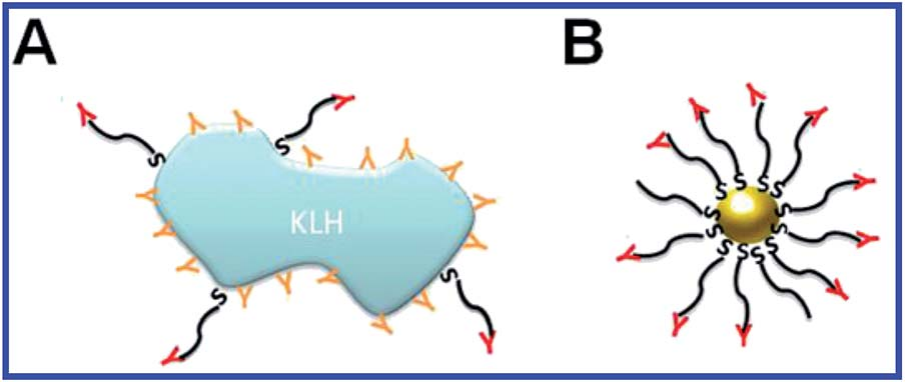
Schematic representation of immunogen localization on the surface of keyhole limpet hemocyanin (KLH) and GNPs, used as antigen carriers. (A) Antibodies toward the peptide–KLH conjugate are produced to the epitopes of both peptide and KLH. (B) Antibodies toward the peptide–GNP conjugate are produced only to the epitopes of the peptide. Reproduced with permission from ref. 116, © 2010, IOP Publishing.

In 1986, Japanese researchers^103^ first reported success in generating antibodies against glutamate by using colloidal gold particles as a carrier. Subsequently, a number of papers were published whose authors applied and further developed this technique to obtain antibodies to the following haptens and complete antigens: amino acids;^104,105^ platelet-activating factor;^106,107^ quinolinic acid;^108^ biotin;^109^ recombinant peptides;^110,111^ lysophosphatide acid;^112^ endostatin;^113^ the capsid peptide of hepatitis C,^114^ influenza,^115^ foot-and-mouth disease,^116,117^ and dengue^118^ viruses; α-amidated peptides;^119^ actin;^120^ antibiotics;^121^ ivermectin;^122,123^ azobenzene;^124^ Aβ-peptide;^125^ clenbuterol;^126^ α-methylacyl-CoA racemase;^127^ *Yersinia*,^128,129^ *Listeria monocytogenes*,^130^ and *Escherichia coli* ^131^ surface antigens; *Neisseria meningitides*,^132^ *Streptococcus pneumoniae*,^133^ and *Burkholderia mallei* ^134,135^ carbohydrate antigens; *Pseudomonas aeruginosa* flagellin;^136^ the transmissible gastroenteritis virus;^29^ tuberculin;^137,138^ the peptides of the malaria plasmodium surface proteins;^139,140^ opisthorchiasis excretory–secretory antigen;^141^ tetanus toxoid.^142^ In all these studies, the haptens or complete antigens were directly conjugated to colloidal gold particles, mixed with complete Freund's adjuvant or alum, and used for animal immunization. As a result, high-titer antisera were obtained that needed no further purification from contaminant antibodies (Fig. 6).

Thus, to date almost 40 publications have demonstrated successful application of functionalized GNPs to obtain antibodies against different antigens. In some cases the application of GNP conjugates produced higher titers and affinity. Often the levels of specific antibodies produced in the immunization of animals with gold nanoparticles conjugated antigens were higher than that generated by classical adjuvants while the amount of antigen required to achieve this response was an order of magnitude lower than for immunization with a standard adjuvant.^143^ The reasons for this may be due to greater accumulation of the antigen in cells such as dendritic cells allowing greater presentation of the therapeutic antigen to the immune system. The readers can find below a similar consideration of a several studies for adjuvant properties of GNPs, although such unique examples is not sufficient to consider a significant massive of collected experimental data.

**Fig. 7:**
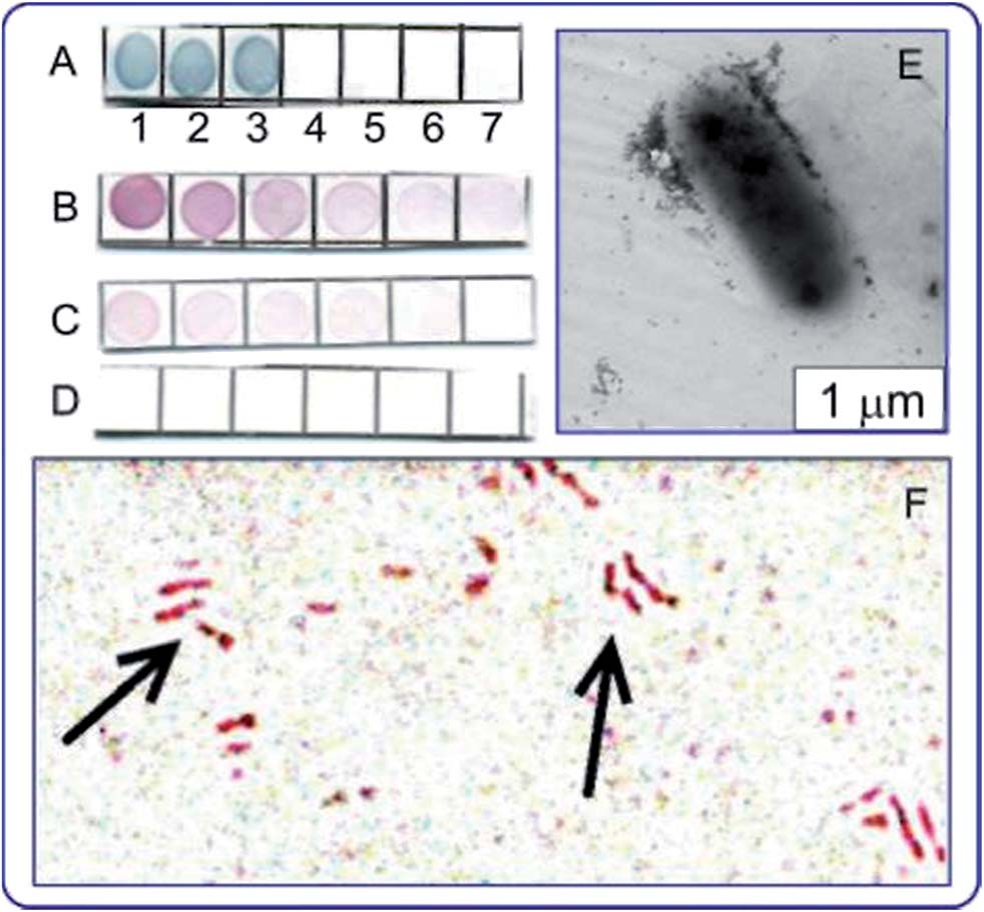
(A) Specificity of antituberculin antibodies as determined by dot analysis using primary labeling with rabbit antituberculin antibodies and secondary labeling with conjugates of antirabbit antibodies with 160/20 nm (SiO_2_ core/Au shell) nanoshells. Sampled antigens: 1 – rabbit anti-tuberculin antibodies; 2 – tuberculin; 3 – *Mycobacteria bovis* BCG; 4 – *Escherichia coli* XL-1 blue; 5 – *Staphylococcus aureus* 209-R; 6 – *Brucella abortus* vaccine strain 82; 7 – brucellin. For samples 1, 2 and 7, the concentrations were 1 mg mL^–1^. (B–D) Dot immunoassay of the mycobacteria *M. bovis* (B), *M. smegmatis* (C) and *M. phlei* (D) by using polyclonal antibodies to tuberculin (primary antibodies) and conjugates of antirabbit antibodies with 15 nm GNPs (secondary antibodies). Note the weak nonspecific coloration of *M. smegmatis* bacteria. (E) TEM image of an *M. bovis* cell treated with antituberculin antibodies and labeled with conjugates of antirabbit antibodies with 15 nm GNPs. The GNP accumulation on the bacterial surface may reflect the localization of the tuberculin antigen. (F) Light microscopy of *M. bovis* BCG treated with rabbit antituberculin antibodies and labeled with conjugates of antirabbit antibodies with 15 nm GNPs. The arrows point to mycobacteria. Reproduced with permission from ref. 138, © 2013, Ivyspring International Publisher.

The use of antituberculin antibodies for immunoassay of mycobacteria described for the first time in ref. 137 and 138. Fig. 7 illustrates applications of the immunodot assay, and TEM and light microscopy imaging to mycobacteria, with the reaction products being visualized by using immunogold markers. In future work, the authors plan to use the GNP + tuberculin conjugates not only to obtain of diagnostic antibodies but also to develop of tuberculin-based anti-tuberculosis vaccines. This can be considered as a new variant of theranostics, which can be called “prophynostics” (prophylaxes + diagnostics).

In 1993, Pow and Crook^144^ suggested attaching a hapten (γ-aminobutyric acid) to a carrier protein before conjugating this complex to colloidal gold. This suggestion was supported in papers devoted to the raising of antibodies to some peptides,^145–149^ amino acids,^150–153^ phenyl-β-d-thioglucoronide,^154^ diminazene.^155^ The antibodies obtained in this way possessed high specificities to the antigens under study and higher (as Pow and Crook^144^ put it, “extremely high”) titers – from 1 : 250 000 to 1 : 1 000 000, as compared with the antibodies produced routinely. At present, the Australian-based company ImmunoSolution offers antibodies, obtained according to ref. 144, to some neurotransmitters and amino acids.

In 1996, Demenev *et al.* ^156^ showed for the first time the possibility of using colloidal gold particles as part of an antiviral vaccine as carriers for the protein antigen of the tick-borne encephalitis virus capsid. According to the authors' data, the offered experimental vaccine had higher protective properties than its commercial analogs, despite the fact that the vaccine did not contain adjuvants.

Subsequently, GNPs have been used to generate antibodies and design experimental vaccines (both peptide and carbohydrate) against influenza A virus,^157,158^ West Nile virus,^159^ the respiratory syncytial virus,^160^ hepatitis E virus,^161^ coronavirus,^162^ as well as against tuberculosis^137^ and listeriosis.^163^ In addition, GNPs are being used in the development of experimental vaccines against tumors^164–170^ and HIV/AIDS.^171–173^ In 2011, Wang *et al.* ^174^ suggested a new therapeutic vaccine based on the combination of myelin-associated inhibitors and GNPs for the treatment of rat medullispinal traumas. Also, for GNP-assisted antigens, several groups reported new administration ways: oral, pulmonary, transcutaneous and transmucosal immunization.^175–180^ Table 2 summarizes the literature data on the antigens and haptens that have been conjugated with GNP carriers and then used for immunization of animals. The titers of the antibodies have been increased owing to GNPs.

**Table 2:** Conjugates of GNPs with antigens and haptens used for immunization and vaccination of animals

Amino acids	Neurotransmitters and hormones	Antibiotics and other drugs	Bacterial, protozoan and viral antigens	Other substances
Glutamate	Acetylcholine	Chloramphenicol	*Yersinia pseudotuberculosis*	Platelet-activating factor
Aspartate	Serotonin	Gentamicin	*Yersinia pestis*	Quinolinic acid
Glycine	Norepinephrine	Neomycin	*Salmonella typhimurium*	Biotin
Serine	Histamine	Lincomycin	*Brucella abortus*	Lysophosphatide acid
Cysteine	Testosterone	Kanamycin	*Mycobacterium tuberculosis*	Immunophilin
Taurine	γ-Aminobutyric acid	Clindamycin	*Streptococcus pneumoniae*	Endostatin
Citrulline	Nortestosterone	Ofloxacinum	*Neisseria meningitides*	Azobenzene
	Estradiol	Tilmicosin	*Burkholderia mallei*	Phenyl-β-d-thioglucoronide
		Ivermectin	*Escherichia coli*	Indole-3-acetic acid
		Diminazene	*Listeria monocytogenes*	Bacteriorhodopsin
		Clenbuterol	*Pseudomonas aeruginosa*	Actin
		Xylazine	*Plasmodium malariae*	Bovine serum albumin
			*Plasmodium falciparum*	Ferritin
			*Opisthorchis felineus*	Tuberculin
			Hepatitis C virus	Tetanus toxoid
			Hepatitis B virus	α-Methylacyl-CoA racemase
			Hepatitis E virus	Protein kinase
			Influenza virus	Carbonic anhydrase
			Foot-and-mouth disease virus	Tumor antigens
			Transmissible gastroenteritis virus	Recombinant and natural peptides
			Tick-borne encephalitis virus	Oligosaccharides
			West Nile virus	
			Respiratory syncytial virus	
			Rabies virus	
			Dengue virus	
			Dengue virus	
			Coronavirus	
			HIV-1	

A considerable number of papers devoted to the use of GNPs for creating DNA vaccines have emerged as well. The principle of DNA immunization is as follows: gene constructions coding for the proteins to which one needs to obtain antibodies are introduced into an organism. If the gene expression is effective, these proteins serve as antigens for the development of an immune response.^181,182^ In the early papers, immunization was conducted by a subcutaneous or intramuscular injection of a “naked” DNA. However, for this purpose, a “biolistic” transfection, using GNPs, began to be applied almost simultaneously. It was found to be very effective, apparently because of the multiplicity of sites of transgene interaction with tissues and because of transgene penetration directly into cells and nuclei.^183,184^ The method of gene immunization, often called DNA vaccination, which was well-developed in experiments with animals, has shown high efficiency especially in respect of viral infections: tick-borne encephalitis, HIV infection, hepatitis B, and some others.^185^

DNA immunization has some advantages over routine vaccination. A single recombinant vector can govern the synthesis of several antigens simultaneously, reducing the number of separate immunizations. This results in erasing problems connected with the difficulties of protein penetration into the organism and in reducing significantly the risk of side effects, which depend on the toxicity of the contaminant proteins introduced during a routine immunization or on the virulence of the bacteria and viruses used. One can expect that DNA immunization will be among the most effective gene-therapy methods in the coming years.^186–188^

Recently, intramuscular injection of a “naked” DNA was abandoned in DNA vaccination. Investigators have come to use nanoparticles as a carrier for genetic material and to introduce the injection substance subcutaneously, intracutaneously, epicutaneously and intranasally.^189–191^ Among the nanoparticles used as DNA carriers, GNPs, both spherical and cylindrical (multivalent Au–Ni nanorods), are especially popular with researchers.^192–198^ Besides DNA, polysaccharides, peptides and glycopeptides are used as vectors in such vaccines.^53,199–205^ Moreover, whereas gold was earlier used only as a carrier, Zhao *et al.* ^206^ noted: “Although the mechanism behind this is not well understood, it appears that gold cartridges might enhance immune responses *in vivo*”.

## 4. Adjuvant properties of gold nanoparticles

Dykman *et al.* ^121,207–209^ proposed a technology for the preparation of antibodies to various antigens, which uses colloidal gold as a carrier and adjuvant. In their method, antigens are adsorbed directly on the GNP surface, with no cross-linking reagents. It was found that animal immunization with colloidal gold–antigen conjugates (with or without the use of Freund's complete adjuvant) yielded specific, high-titer antibodies to a variety of antigens, with no concomitant antibodies. GNPs can stimulate antibody synthesis in rabbits, rats and mice, and the amount of antigen required is reduced, as compared with that needed with some conventional adjuvant (Table 3).

**Table 3:** The antibody titers obtained during immunization of rabbits with *Yersinia* antigen

Preparation	1st immunization	2nd immunization	Boosting
Colloidal gold + antigen (1 mg)	1 : 32	1 : 256	1 : 10 240
Complete Freund's adjuvant + antigen (100 mg)	1 : 32	1 : 256	1 : 10 240
Physiological saline + antigen (100 mg)	1 : 2	1 : 16	1 : 512

In summary, the experimental results give grounds to state that:

(1) Using the method of “gold immunization,” one can obtain antibodies to those haptens to which it is very difficult to obtain antibodies conventionally (in particular, several antibiotics, vitamins and nonimmunogenic peptides);

(2) The amount of antigen used for immunization in this case is much smaller than that used in conventional methods, even when the latter allow one to obtain an immune response;

(3) In the experiments with several antigens conjugated with GNPs, an immune response was obtained without the use of other adjuvants;

(4) GNPs used as an antigen carrier stimulate the phagocytic activity of lymphoid cells and induce the release of inflammatory mediators.

All the above facts show decisively that GNPs possesses adjuvant properties. With use of GNPs as an antigen carriers they activated the phagocytic activity of macrophages and influenced the functioning of lymphocytes (see above), which apparently may be responsible for their immunomodulating effect. It also was found that GNPs and their conjugates with low- and high-molecular weight antigens stimulate the respiratory activity of cells of the reticuloendothelial system and the activity of macrophage mitochondrial enzymes,^28^ which possibly determines the adjuvant properties of colloidal gold. That GNPs act as both an adjuvant and a carrier (*i.e.*, they present haptens to T cells) seems the most interesting aspect of manifestation of immunogenic properties by colloidal gold. In particular, GNPs conjugated to antigens were found to influence the activation of T cells: a tenfold increase in proliferation, as compared with that observed on the addition of the native antigen, was found. This fact shows that there is a fundamental possibility of targeted activation of T cells followed by macrophage activation and pathogen killing.

Several authors have reported a successful therapy of rheumatoid arthritis with a colloidal gold solution.^210–213^ According to the data of Graham,^214^ the effect of GNPs in this case is an inhibition of monocyte-induced lymphocyte proliferation. The transformation of Au(0) to Au(i) in the immune-system cells under the action of several amino acids was discussed by Merchant.^215^ It was noted by Eisler^216^ that injection of GNPs into laboratory animals could result in an inflammatory response, accumulation of gold in the reticular cells of lymphoid tissue, and activation of cellular and humoral immunity.

However, not a single paper available to us has reported data on the mechanism of such properties of gold particles. In our opinion, the reasoning given by Pow and Crook^144^ on the preferable macrophage response to corpuscular antigens, as opposed to soluble ones, is certainly valid. This fact has also been confirmed by researchers studying the mechanism of action of DNA vaccines and using gold particles to deliver genetic material to cells.^206^ The role of Kupffer and Langerhans cells in the development of immune response was shown in those investigations. The influence of dendritic cells on the development of immune response upon injection of a GNP-conjugated antigen was discussed by Vallhov *et al.* ^217^ In addition, those authors noted that when using nanoparticles in medical practice, one has to ensure that there are no lipopolysaccharides on their surface. Similar results, for the interaction of GNPs with macrophages, were reported by Kingston *et al.* ^218^ The interaction of cells of the immune system with GNPs was very actively examined by Dobrovolskaia's group.^72,73,75,102,219,220^ According to them, nanoimmunology is a new promising and rapidly developing field. In spite of the many obstacles, significant progress in our understanding of nanoparticle interaction with the components of the immune system has been achieved. However, much is yet to be studied and understood.

Modern trends in the use of GNPs for vaccination is the application of multivalent glycopolymers^202^ and peptides;^57^ combined use of GNPs and other immunostimulants, in particular CpG (including as conjugated with GNPs),^36,38,221–226^ polyvalent nucleic acid,^39,227^ and plant adjuvants, *e.g.*, extracts from *Quillaja saponaria*,^228^ *Asparagus racemosus* ^229^ or *Tamarindus indica*;^230^ and the application of GNPs of various sizes and shapes (including nanorods, nanocubic, nanocages, nanoclusters).^159,231–233^

**Fig. 8:**
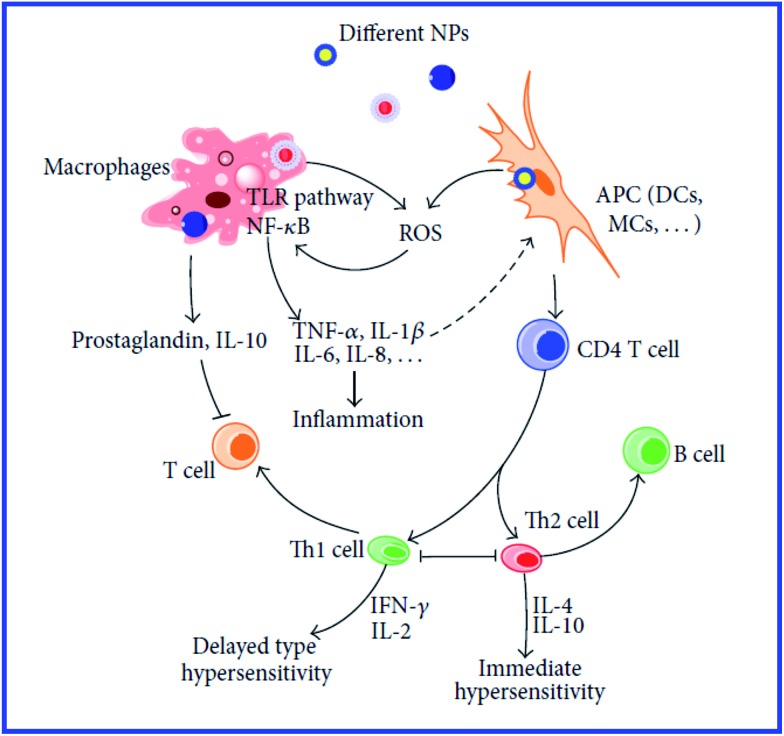
Mechanisms involved in NP-induced immunomodulation. The stimulation/suppression of the immune system depends on the nature of the NPs and results in different outcomes. NPs, nanoparticles; NF-κB, nuclear factor kappa B; TLR pathway: toll-like receptor pathway; APC, antigen-presenting cell; DCs, dendritic cells; MCs, mast cells; GM-CSF, granulocyte-macrophage colony-stimulating factor; Th0, type 0 T-helper lymphocyte; Th1, type 1 T-helper lymphocyte; Th2, type 2 T-helper lymphocyte; solid line with arrow, activate/release/induce; solid line with vertical dashes at ends, inhibit; dotted line, possible influence; broken line, polarization/differentiation. Reproduced with permission from ref. 234, © 2014, Hindawi Publishing Corporation.

**Fig. 9:**
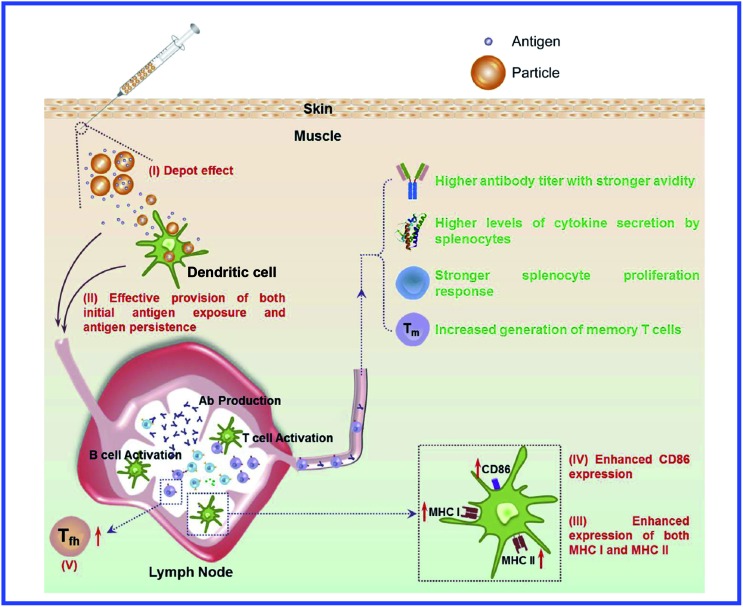
Schematic illustration of the proposed mode of action of the combined vaccine formulation composed of nanoparticles-encapsulated antigen and soluble antigen mixed with blank nanoparticles. Reproduced with permission from ref. 235, © 2014, Elsevier.

However, those data do not answer the question about the further mechanisms of antigen presentation to T helpers. According to the current view,^94^ the presentation of an antigen to T cells is preceded by its processing, *i.e.*, cleavage into peptide fragments followed by the formation of bonds with molecules of the major histocompatibility complex, which transport the antigen fragment to the surface of the antigen-presenting cell. It remains unclear, then, how this process can proceed with a hapten. The hypothesis of the multivalent antigen, *i.e.*, the antigen formed because of the high local concentration of univalent antigens on the surface of a gold particle, does not answer this question either. Hypothetical mechanisms of the immunomodulatory effects of nanoparticles are shown in Fig. 8 and 9.^234,235^

Recently, many papers have been published in which the problems associated with GNP use for targeted drug delivery were discussed. In our opinion, one should deal with this question very carefully, taking into account the possibility of production in animals or humans of antibodies specific to the administered drug adsorbed on gold particles. We believe that the discovery of adjuvant properties of GNPs creates favorable conditions for designing next-generation vaccines.

Alongside GNPs, other nonmetallic nanoparticles also can serve as antigen carriers. The published examples include liposomes, proteosomes, microcapsules, fullerenes, carbon nanotubes, dendrimers and paramagnetic particles.^208^ In our view, especially promising carriers are synthetic and natural polymeric biodegradable nanomaterials [polymethyl methacrylate, poly(lactide-*co*-glycolid acid), chitosan, gelatin]. With the use of such nanoparticles, the immunogenicity of a loaded substance and its representation in a host immune system will be changed. A nanoparticle conjugate with an absorbed or a capsulated antigen can serve as an adjuvant for the optimization of immune response after vaccination.

The evident advantages of biodegradable nanoparticles is their complete utilization in the vaccinated organism, high loading efficiency for the target substance, enhanced ability to cross various physiological barriers, and low systemic side effects. In all likelihood, the immune action of biodegradable nanoparticles and GNPs as corpuscular carriers are similar. Keeping in mind the recent data for the low toxicity of GNPs and their efficient excretion by the hepatobiliary system, we expect that both nanoparticle classes – GNPs and biodegradable nanoparticles – will compete on equal footing for the development of next-generation vaccines.

## 5. Conclusions

Thus, GNP uptake into cells of the immune system activates the production of proinflammatory cytokines, a finding which indicates directly that GNPs are immunostimulatory. The activation of immune cells by GNPs, shown by several authors, may serve as a basis to develop new vaccine adjuvants. As in the case of the usual cells, interactions with various types of receptors on the surface of immune cells and, correspondingly, various types of GNP endocytosis depend largely on the surface functionalization of GNPs. Many researchers believe that the key role in macrophage uptake of GNPs is played by scavenger receptors. However, the interaction of functionalized GNPs with cells of the immune system is still far from being understood in more or less detail and requires further study.^236^

In conclusion, it may be said that the time is probably right to talk of not only the biochemistry but also the biophysics of immune response, because it is the unique biophysical properties of metallic particles—in particular, the surface charge and the electrostatic field of the particle (influencing, in a certain manner, the charge, orientation and polarization of the antigen molecules adsorbed on the particles)—that have to significantly affect the immune-response process.

Thus, the GNPs can serve as adjuvants to improve the effectiveness of vaccines, stimulate antigen-presenting cells, and provide controlled release of antigens. In addition, the immunogenicity of CNPs is determined by the physicochemical properties of particles such as size, shape, charge and surface functionalization. Study of the immune response characteristics when using GNPs as a carrier and adjuvant for the production of antibodies will allow evaluating their potential for the development of effective vaccines.

## References

[cit1] Sapsford K. E., Algar W. R., Berti L., Gemmill K. B., Casey B. J., Oh E., Stewart M. H., Medintz I. L. (2013). Chem. Rev..

[cit2] Khlebtsov N. G., Dykman L. A. (2010). J. Quant. Spectrosc. Radiat. Transfer.

[cit3] Handbook of Immunological Properties of Engineered Nanomaterials, ed. M. A. Dobrovolskaia and S. E. McNeil, World Scientific Publ., Singapore, 2013.

[cit4] Boisselier E., Astruc D. (2009). Chem. Soc. Rev..

[cit5] Dykman L. A., Khlebtsov N. G. (2012). Chem. Soc. Rev..

[cit6] Dreaden E. C., Alkilany A. M., Huang X., Murphy C. J., El-Sayed M. A. (2012). Chem. Soc. Rev..

[cit7] Brown C. L., Whitehouse M. W., Tiekink E. R. T., Bushell G. R. (2008). Inflammopharmacology.

[cit8] Scott G. B., Williams H. S., Marriott P. M. (1967). Br. J. Exp. Pathol..

[cit9] Singer J. M., Adlersberg L., Sadek M. (1972). J. Reticuloendothel. Soc..

[cit10] Hardonk M. J., Harms G., Koudstaal J. (1985). Histochemistry.

[cit11] Renaud G., Hamilton R. L., Havel R. (1989). Hepatology.

[cit12] Sadauskas E., Wallin H., Stoltenberg M., Vogel U., Doering P., Larsen A., Danscher G. (2007). Part. Fibre Toxicol..

[cit13] Sadauskas E., Danscher G., Stoltenberg M., Vogel U., Larsen A., Wallin H. (2009). Nanomed.: Nanotechnol., Biol. Med..

[cit14] Khlebtsov N. G., Dykman L. A. (2011). Chem. Soc. Rev..

[cit15] Dykman L. A., Khlebtsov N. G. (2014). Chem. Rev..

[cit16] Shukla R., Bansal V., Chaudhary M., Basu A., Bhonde R. R., Sastry M. (2005). Langmuir.

[cit17] Yen H.-J., Hsu S.-h., Tsai C.-L. (2009). Small.

[cit18] Lim Y. T., Cho M. Y., Choi B. S., Noh Y.-W., Chung B. H. (2008). Nanotechnology.

[cit19] Zhang Q., Hitchins V. M., Schrand A. M., Hussain S. M., Goering P. L. (2011). Nanotoxicology.

[cit20] Sumbayev V. V., Yasinska I. M., Garcia C. P., Gilliland D., Lall G. S., Gibbs B. F., Bonsall D. R., Varani L., Rossi F., Calzolai L. (2013). Small.

[cit21] le Guevél X., Palomares F., Torres M. J., Blanca M., Fernandez T. D., Mayorga C. (2015). RSC Adv..

[cit22] Choi M.-R., Stanton-Maxey K. J., Stanley J. K., Levin C. S., Bardhan R., Akin D., Badve S., Sturgis J., Robinson J. P., Bashir R., Halas N. J., Clare S. E. (2007). Nano Lett..

[cit23] Dreaden E. C., Mwakwari S. C., Austin L. A., Kieffer M. J., Oyelere A. K., El-Sayed M. A. (2012). Small.

[cit24] Tian Y., Cui Y., Lou H., Li J., Yan P. (2007). Chinese Agricultural Science Bulletin.

[cit25] Lou H., Tian Y., Gao J.-Q., Deng S.-Y., Li J.-L. (2007). J. Foshan Univ., Nat. Sci. Ed..

[cit26] Bastús N. G., Sánchez-Tilló E., Pujals S., Farrera C., Kogan M. J., Giralt E., Celada A., Lloberas J., Puntes V. (2009). Mol. Immunol..

[cit27] Bastús N. G., Sánchez-Tilló E., Pujals S., Farrera C., López C., Kogan M. J., Giralt E., Celada A., Lloberas J., Puntes V. (2009). ACS Nano.

[cit28] Staroverov S. A., Aksinenko N. M., Gabalov K. P., Vasilenko O. A., Vidyasheva I. V., Shchyogolev S. Y., Dykman L. A. (2009). Gold Bull..

[cit29] Staroverov S. A., Vidyasheva I. V., Gabalov K. P., Vasilenko O. A., Laskavyi V. N., Dykman L. A. (2011). Bull. Exp. Biol. Med..

[cit30] Lee J. Y., Park W., Yi D. K. (2012). Toxicol. Lett..

[cit31] Xu L., Liu Y., Chen Z., Li W., Liu Y., Wang L., Liu Y., Wu X., Ji Y., Zhao Y., Ma L., Shao Y., Chen C. (2012). Nano Lett..

[cit32] Zlobina O. V., Bugaeva I. O., Maslyakova G. N., Firsova S. S., Bucharskaya A. B., Khlebtsov N. G., Khlebtsov B. N., Dykman L. A. (2012). Russian Open Medical Journal.

[cit33] Brown D. M., Johnston H., Gubbins E., Stone V. (2014). J. Biomed. Nanotechnol..

[cit34] Bancos S., Stevens D. L., Tyner K. M. (2015). Int. J. Nanomed..

[cit35] Fallarini S., Paoletti T., Battaglini C. O., Ronchi P., Lay L., Bonomi R., Jha S., Mancin F., Scrimin P., Lombardi G. (2013). Nanoscale.

[cit36] Wei M., Chen N., Li J., Yin M., Liang L., He Y., Song H., Fan C., Huang Q. (2012). Angew. Chem., Int. Ed..

[cit37] Rothenfusser S., Tuma E., Wagner M., Endres S., Hartmann G. (2003). Curr. Opin. Mol. Ther..

[cit38] Tsai C.-Y., Lu S.-L., Hu C.-W., Yeh C.-S., Lee G.-B., Lei H.-Y. (2012). J. Immunol..

[cit39] Massich M. D., Giljohann D. A., Seferos D. S., Ludlow L. E., Horvath C. M., Mirkin C. A. (2009). Mol. Pharmaceutics.

[cit40] Kim E.-Y., Schulz R., Swantek P., Kunstman K., Malim M. H., Wolinsky S. M. (2012). Gene Ther..

[cit41] Walkey C. D., Olsen J. B., Guo H., Emili A., Chan W. C. W. (2012). J. Am. Chem. Soc..

[cit42] Lynch I., Dawson K. A. (2008). Nano Today.

[cit43] Nel A. E., Mädler L., Velegol D., Xia T., Hoek E. M. V., Somasundaran P., Klaessig F., Castranova V., Thompson M. (2009). Nat. Mater..

[cit44] Dobrovolskaia M. A., Patri A. K., Zheng J., Clogston J. D., Ayub N., Aggarwal P., Neun B. W., Hall J. B., McNeil S. E. (2009). Nanomed.: Nanotechnol., Biol. Med..

[cit45] Lacerda S. H. D. P., Park J. J., Meuse C., Pristinski D., Becker M. L., Karim A., Douglas J. F. (2010). ACS Nano.

[cit46] Braun N. J., DeBrosse M. C., Hussain S. M., Comfort K. K. (2016). Mater. Sci. Eng., C.

[cit47] Sasidharan A., Chandran P., Monteiro-Riviere N. A. (2016). ACS Biomater. Sci. Eng..

[cit48] Ma J. S., Kim W. J., Kim J. J., Kim T. J., Ye S. K., Song M. D., Kang H., Kim D. W., Moon W. K., Lee K. H. (2010). Nitric Oxide.

[cit49] Liu Z., Li W., Wang F., Sun C., Wang L., Wang J., Sun F. (2012). Nanoscale.

[cit50] Goldstein A., Soroka Y., Frusic-Zlotkin M., Lewis A., Kohen R. (2016). Nanoscale.

[cit51] García C. P., Sumbayev V., Gilliland D., Yasinska I. M., Gibbs B. F., Mehn D., Calzolai L., Rossi F. (2013). Sci. Rep..

[cit52] Ueno H., Klechevsky E., Morita R., Aspord C., Cao T., Matsui T., Di Pucchio T., Connolly J., Fay J. W., Pascual V., Palucka A. K., Banchereau J. (2007). Immunol. Rev..

[cit53] Cheung W.-H., Chan V. S.-F., Pang H.-W., Wong M.-K., Guo Z.-H., Tam P. K.-H., Che C.-M., Lin C.-L., Yu W.-Y. (2009). Bioconjugate Chem..

[cit54] Cruz L. J., Rueda F., Cordobilla B., Simón L., Hosta L., Albericio F., Domingo J. C. (2011). Mol. Pharmaceutics.

[cit55] Villiers C. L., Freitas H., Couderc R., Villiers M.-B., Marche P. N. (2010). J. Nanopart. Res..

[cit56] Ye F., Vallhov H., Qin J., Daskalaki E., Sugunan A., Toprak M. S., Fornara A., Gabrielsson S., Scheynius A., Muhammed M. (2011). Int. J. Nanotechnol..

[cit57] Lin A. Y., Lunsford J., Bear A. S., Young J. K., Eckels P., Luo L., Foster A. E., Drezek R. A. (2013). Nanoscale Res. Lett..

[cit58] Fytianos K., Rodriguez-Lorenzo L., Clift M. J., Blank F., Vanhecke D., von Garnier C., Petri-Fink A., Rothen-Rutishauser B. (2015). Nanomed.: Nanotechnol., Biol. Med..

[cit59] Małaczewska J. (2015). Pol. J. Vet. Sci..

[cit60] Małaczewska J. (2015). Pol. J. Vet. Sci..

[cit61] Sharma M., Salisbury R. L., Maurer E. I., Hussain S. M., Sulentic C. E. W. (2013). Nanoscale.

[cit62] Lee C.-H., Syu S.-H., Chen Y.-S., Hussain S. M., Onischuk A. A., Chen W. L., Huang G. S. (2014). Nanotechnology.

[cit63] Liptrott N. J., Kendall E., Nieves D. J., Farrell J., Rannard S., Fernig D. G., Owen A. (2014). Nanomedicine.

[cit64] Cho W.-S., Cho M., Jeong J., Choi M., Cho H.-Y., Han B. S., Kim S. H., Kim H. O., Lim Y. T., Chung B. H., Jeong J. (2009). Toxicol. Appl. Pharmacol..

[cit65] Bartneck M., Keul H. A., Singh S., Czaja K., Bornemann J., Bockstaller M., Möller M., Zwadlo-Klarwasser G., Groll J. (2010). ACS Nano.

[cit66] Bartneck M., Keul H. A., Zwadlo-Klarwasser G., Groll J. (2010). Nano Lett..

[cit67] Bartneck M., Keul H. A., Wambach M., Bornemann J., Gbureck U., Chatain N., Neuss S., Tacke F., Groll J., Zwadlo-Klarwasser G. (2012). Nanomed.: Nanotechnol., Biol. Med..

[cit68] DeFranco A. L., Locksley R. M. and Robertson M., Immunity: The Immune Response to Infection, Oxford University Press, Oxford, 2007.

[cit69] Blander J. M., Medzhitov R. (2006). Nat. Immunol..

[cit70] Deng Z. J., Liang M., Monteiro M., Toth I., Minchin R. F. (2011). Nat. Nanotechnol..

[cit71] Arnáiz B., Martinez-Ávila O., Falcon-Perez J. M., Penadés S. (2012). Bioconjugate Chem..

[cit72] Dobrovolskaia M. A., McNeil S. E. (2007). Nat. Nanotechnol..

[cit73] Dobrovolskaia M. A., Aggarwal P., Hall J. B., McNeil S. E. (2008). Mol. Pharmaceutics.

[cit74] Patel P. C., Giljohann D. A., Daniel W. L., Zheng D., Prigodich A. E., Mirkin C. A. (2010). Bioconjugate Chem..

[cit75] França A., Aggarwal P., Barsov E. V., Kozlov S. V., Dobrovolskaia M. A., González-Fernández Á. (2011). Nanomedicine.

[cit76] Zilber L. A., Friese W. W. (1929). Zh. Eksp. Biol. Med..

[cit77] Huang G. S., Chen Y.-S., Yeh H.-W. (2006). Nano Lett..

[cit78] Steabben D. B. (1925). Br. J. Exp. Pathol..

[cit79] Zozaya J., Clark J. (1933). J. Exp. Med..

[cit80] Pacheco G. (1925). Mem. Inst. Oswaldo Cruz.

[cit81] Gros O., O'Connor J. M. (1911). Naunyn-Schmiedebergs Arch. Pharmacol..

[cit82] Stills Jr H. F. (2005). ILAR J..

[cit83] Reed S. G., Orr M. T., Fox C. B. (2013). Nat. Med..

[cit84] Zhang X.-D., Wu D., Shen X., Liu P.-X., Yang N., Zhao B., Zhang H., Sun Y.-M., Zhang L.-A., Fan F.-Y. (2011). Int. J. Nanomed..

[cit85] Bucharskaya A. B., Pakhomy S. S., Zlobina O. V., Maslyakova G. N., Matveeva O. V., Bugaeva I. O., Navolokin N. A., Khlebtsov B. N., Bogatyrev V. A., Khlebtsov N. G., Tuchin V. V. (2016). J. Innovative Opt. Health Sci..

[cit86] Yu Q., Li J., Zhang Y., Wang Y., Liu L., Li M. (2016). Sci. Rep..

[cit87] Kovalev I. E. and Polevaya O. Y., Biochemical Foundations of Immunity to Low-Molecular Chemical Compounds, Nauka, Moscow, 1985, in Russian.

[cit88] Arnon R., Horwitz R. J. (1992). Curr. Opin. Immunol..

[cit89] Ben-Yedidia T., Arnon R. (1997). Curr. Opin. Biotechnol..

[cit90] Bloom B. R. and Lambert P.-H., The Vaccine Book, Academic Press, San Diego, CA, 2003.

[cit91] Moisa A. A., Kolesanova E. F. (2010). Biochem. Suppl. Ser. B: Biomed. Chem..

[cit92] Li W., Joshi M. D., Singhania S., Ramsey K. H., Murthy A. K. (2014). Vaccines.

[cit93] Vartak A., Sucheck S. J. (2016). Vaccines.

[cit94] Male D., Brostoff J., Roth D. and Roitt I., Immunology, Saunders, Philadelphia, 2012.

[cit95] Kumar B. S., Ashok V., Kalyani P., Nair G. R. (2016). Veterinary World.

[cit96] Petrov R. V. and Khaitov R. M., Immunogenes and vaccines of new generation, GEOTAR-Media, Moscow, 2011, in Russian.

[cit97] Zaman M., Good M. F., Toth I. (2013). Methods.

[cit98] Prashant C. K., Kumar M., Dinda A. K. (2014). J. Biomed. Nanotechnol..

[cit99] Aklakur M., Rather M. A., Kumar N. (2016). Crit. Rev. Food Sci. Nutr..

[cit100] Salazar-González J. A., González-Ortega O., Rosales-Mendoza S. (2015). Expert Rev. Vaccines.

[cit101] Gupta A., Das S., Schanen B., Seal S. (2016). Wiley Interdiscip. Rev.: Nanomed. Nanobiotechnol..

[cit102] Ilinskaya A. N., Dobrovolskaia M. A. (2016). Toxicol. Appl. Pharmacol..

[cit103] Shiosaka S., Kiyama H., Wanaka A., Tohyama M. (1986). Brain Res..

[cit104] Wanaka A., Shiotani Y., Kiyama H., Matsuyama T., Kamada T., Shiosaka S., Tohyama M. (1987). Exp. Brain Res..

[cit105] Ottersen O. P., Storm-Mathisen J. (1987). Trends Neurosci..

[cit106] Tomii A., Masugi F. (1991). Jpn. J. Med. Sci. Biol..

[cit107] Tatsumi N., Terano Y., Hashimoto K., Hiyoshi M., Matsuura S. (1993). Osaka City Med. J..

[cit108] Moffett J. R., Espey M. G., Namboodiri M. A. A. (1994). Cell Tissue Res..

[cit109] Dykman L. A., Matora L. Y., Bogatyrev V. A. (1996). J. Microbiol. Methods.

[cit110] Walensky L. D., Gascard P., Fields M. E., Blackshaw S., Conboy J. G., Mohandas N., Snyder S. H. (1998). J. Cell Biol..

[cit111] Walensky L. D., Dawson T. M., Steiner J. P., Sabatini D. M., Suarez J. D., Klinefelter G. R., Snyder S. H. (1998). Mol. Med..

[cit112] Chen J., Zou F., Wang N., Xie S., Zhang X. (2000). Bioorg. Med. Chem. Lett..

[cit113] Feldman A. L., Tamarkin L., Paciotti G. F., Simpson B. W., Linehan W. M., Yang J. C., Fogler W. E., Turner E. M., Alexander H. R., Libutti S. K. (2000). Clin. Cancer Res..

[cit114] Olenina L. V., Kolesanova E. F., Gervaziev Y. V., Zaitseva I. S., Kuraeva T. E., Sobolev B. N., Archakov A. I. (2001). Med. Immunol..

[cit115] Chen Y.-S., Hung Y.-C., Liau I., Huang G. S. (2009). Nanoscale Res. Lett..

[cit116] Chen Y.-S., Hung Y.-C., Liau I., Huang G. S. (2010). Nanotechnology.

[cit117] Dykman L. A., Staroverov S. A., Mezhenny P. V., Fomin A. S., Kozlov S. V., Volkov A. A., Laskavy V. N., Shchyogolev S. Y. (2015). Gold Bulletin.

[cit118] Versiani A. F., Andrade L. M., Martins E. M. N., Scalzo S., Geraldo J. M., Chaves C. R., Ferreira D. C., Ladeira M., Guatimosim S., Ladeira L. O., da Fonseca F. G. (2016). Future Virol..

[cit119] Mueller G. P. and Driscoll W. J., in Posttranslational Modification of Proteins: Tools for Functional Proteomics, ed. C. Kannicht, Humana Press, Totowa, 2002, pp. 241–257.

[cit120] Dykman L. A., Bogatyrev V. A., Zaitseva I. S., Sokolova M. K., Ivanov V. V., Sokolov O. I. (2002). Biophysics.

[cit121] Dykman L. A., Sumaroka M. V., Staroverov S. A., Zaitseva I. S., Bogatyrev V. A. (2004). Biology Bulletin.

[cit122] Staroverov S. A., Pristensky D. V., Yermilov D. N., Semenov S. V., Aksinenko N. M., Shchyogolev S. Y., Dykman L. A. (2007). Biotechnology.

[cit123] Pristensky D. V., Staroverov S. A., Ermilov D. N., Shchyogolev S. Y., Dykman L. A. (2007). Biochem. Suppl. Ser. B: Biomed. Chem..

[cit124] Ishii N., Fitrilawati F., Manna A., Akiyama H., Tamada Y., Tamada K. (2008). Biosci., Biotechnol., Biochem..

[cit125] Kayed R., Head E., Thompson J. L., McIntire T. M., Milton S. C., Cotman C. W., Glabe C. G. (2003). Science.

[cit126] Vasilenko O. A., Staroverov S. A., Yermilov D. N., Pristensky D. V., Shchyogolev S. Y., Dykman L. A. (2007). Immunopharmacol. Immunotoxicol..

[cit127] Dykman L. A., Staroverov S. A., Fomin A. S., Panfilova E. V., Shirokov A. A., Bucharskaya A. B., Maslyakova G. N., Khlebtsov N. G. (2016). Gold Bulletin.

[cit128] Staroverov S. A., Ermilov D. N., Shcherbakov A. A., Semenov S. V., Shchyegolev S. Y., Dykman L. A. (2003). Zh. Mikrobiol., Epidemiol. Immunobiol..

[cit129] Gregory A. E., Williamson E. D., Prior J. L., Butcher W. A., Thompson I. J., Shaw A. M., Titball R. W. (2012). Vaccine.

[cit130] Rodriguez-Del Rio E., Marradi M., Calderon-Gonzalez R., Frande-Cabanes E., Penadés S., Petrovsky N., Alvarez-Dominguez C. (2015). Vaccine.

[cit131] Gao W., Fang R. H., Thamphiwatana S., Luk B. T., Li J., Angsantikul P., Zhang Q., Hu C.-M. J., Zhang L. (2015). Nano Lett..

[cit132] Manea F., Bindoli C., Fallarini S., Lombardi G., Polito L., Lay L., Bonomi R., Mancin F., Scrimin P. (2008). Adv. Mater..

[cit133] Safari D., Marradi M., Chiodo F., Dekker H. A. T., Shan Y., Adamo R., Oscarson S., Rijkers G. T., Lahmann M., Kamerling J. P., Penadés S., Snippe H. (2012). Nanomedicine.

[cit134] Gregory A. E., Judy B. M., Qazi O., Blumentritt C. A., Brown K. A., Shaw A. M., Torres A. G., Titball R. W. (2015). Nanomed.: Nanotechnol., Biol. Med..

[cit135] Torres A. G., Gregory A. E., Hatcher C. L., Vinet-Oliphant H., Morici L. A., Titball R. W., Roy C. J. (2015). Vaccine.

[cit136] Dakterzada F., Mohabati Mobarez A., Habibi Roudkenar M., Mohsenifar A. (2016). Vaccine.

[cit137] Staroverov S. A., Dykman L. A. (2013). Nanotechnology.

[cit138] Khlebtsov N. G., Bogatyrev V. A., Dykman L. A., Khlebtsov B. N., Staroverov S. A., Shirokov A. A., Matora L. Y., Khanadeev V. A., Pylaev T. E., Tsyganova N. A., Terentyuk G. S. (2013). Theranostics.

[cit139] Parween S., Gupta P. K., Chauhan V. S. (2011). Vaccine.

[cit140] Kumar R., Ray P. C., Datta D., Bansal G. P., Angov E., Kumar N. (2015). Vaccine.

[cit141] Bulashev A. K., Serikova S. S., Eskendirova S. Z. (2014). Biotechnology. Theory and practice..

[cit142] Barhate G. A., Gaikwad S. M., Jadhav S. S., Pokharkar V. B. (2014). Int. J. Pharm..

[cit143] David C. A. W., Owen A., Liptrott N. J. (2016). Nanomedicine.

[cit144] Pow D. V., Crook D. K. (1993). J. Neurosci. Methods.

[cit145] Baude A., Nusser Z., Molnár E., McIlhinney R. A. J., Somogyi P. (1995). Neuroscience.

[cit146] Harris D. P., Vordermeier H.-M., Arya A., Bogdan K., Moreno C., Ivanyi J. (1996). Immunology.

[cit147] Pickard L., Noël J., Henley J. M., Collingridge G. L., Molnar E. (2000). J. Neurosci..

[cit148] Schäfer M. K.-H., Varoqui H., Defamie N., Weihe E., Erickson J. D. (2002). J. Biol. Chem..

[cit149] Holmseth S., Dehnes Y., Bjørnsen L. P., Boulland J.-L., Furness D. N., Bergles D., Danbolt N. C. (2005). Neuroscience.

[cit150] Schell M. J., Molliver M. E., Snyder S. H. (1995). Proc. Natl. Acad. Sci. U. S. A..

[cit151] Schell M. J., Cooper O. B., Snyder S. H. (1997). Proc. Natl. Acad. Sci. U. S. A..

[cit152] Eliasson M. J. L., Blackshaw S., Schell M. J., Snyder S. H. (1997). Proc. Natl. Acad. Sci. U. S. A..

[cit153] Huster D., Hjelle O. P., Haug F.-M., Nagelhus E. A., Reichelt W., Ottersen O. P. (1998). Anat. Embryol..

[cit154] Staimer N., Gee S. J., Hammock B. D. (2001). Fresenius' J. Anal. Chem..

[cit155] Staroverov S. A., Vasilenko O. A., Gabalov K. P., Pristensky D. V., Yermilov D. N., Aksinenko N. M., Shchyogolev S. Y., Dykman L. A. (2008). Int. Immunopharmacol..

[cit156] Demenev V. A., Shchinova M. A., Ivanov L. I., Vorobeva R. N., Zdanovskaia N. I., Nebaikina N. V. (1996). Vopr. Virusol..

[cit157] Mezhenny P. V., Staroverov S. A., Volkov A. A., Kozlov S. V., Laskavy V. N., Dykman L. A., Isayeva A. Y. (2013). Bulletin.

[cit158] Tao W., Ziemer K. S., Gill H. S. (2014). Nanomedicine.

[cit159] Niikura K., Matsunaga T., Suzuki T., Kobayashi S., Yamaguchi H., Orba Y., Kawaguchi A., Hasegawa H., Kajino K., Ninomiya T., Ijiro K., Sawa H. (2013). ACS Nano.

[cit160] Stone J. W., Thornburg N. J., Blum D. L., Kuhn S. J., Wright D. W., Crowe Jr J. E. (2013). Nanotechnology.

[cit161] Wang H., Ding Y., Su S., Meng D., Mujeeb A., Wu Y., Nie G. (2016). Nanoscale Horiz..

[cit162] Chen H.-W., Huang C.-Y., Lin S.-Y., Fang Z.-S., Hsu C.-H., Lin J.-C., Chen Y. I., Yao B.-Y., Hu C.-M. J. (2016). Biomaterials.

[cit163] Calderón-Gonzalez R., Terán-Navarro H., Frande-Cabanes E., Ferrández-Fernández E., Freire J., Penadés S., Marradi M., García I., Gomez-Román J., Yañez-Díaz S., Álvarez-Domínguez C. (2016). Nanomaterials.

[cit164] Almeida J. P. M., Figueroa E. R., Drezek R. A. (2014). Nanomed.: Nanotechnol., Biol. Med..

[cit165] Cao-Milán R., Liz-Marzán L. M. (2014). Expert Opin. Drug Delivery.

[cit166] Lee I.-H., Kwon H.-K., An S., Kim D., Kim S., Yu M. K., Lee J.-H., Lee T.-S., Im S.-H., Jon S. (2012). Angew. Chem., Int. Ed..

[cit167] Park Y.-M., Lee S. J., Kim Y. S., Lee M. H., Cha G. S., Jung I. D., Kang T. H., Han H. D. (2013). Immune Network.

[cit168] Ahn S., Lee I.-H., Kang S., Kim D., Choi M., Saw P. E., Shin E.-C., Jon S. (2014). Adv. Healthcare Mater..

[cit169] Almeida J. P. M., Lin A. Y., Figueroa E. R., Foster A. E., Drezek R. A. (2015). Small.

[cit170] Biswas S., Medina S. H., Barchi Jr J. J. (2015). Carbohydr. Res..

[cit171] Chiodo F., Enríquez-Navas P. M., Angulo J., Marradi M., Penadés S. (2015). Carbohydr. Res..

[cit172] Gianvincenzo P. D., Calvo J., Perez S., Álvarez A., Bedoya L. M., Alcamí J., Penadés S. (2015). Bioconjugate Chem..

[cit173] Liu Y., Chen C. (2016). Adv. Drug Delivery Rev..

[cit174] Wang Y.-T., Lu X.-M., Zhu F., Huang P., Yu Y., Zeng L., Long Z.-Y., Wu Y.-M. (2011). Biomaterials.

[cit175] Marasini N., Skwarczynski M., Toth I. (2014). Expert Rev. Vaccines.

[cit176] Ballester M., Nembrini C., Dhar N., de Titta A., de Piano C., Pasquier M., Simeoni E., van der Vlies A. J., McKinney J. D., Hubbell J. A., Swartz M. A. (2011). Vaccine.

[cit177] Gupta P. N., Vyas S. P. (2011). Curr. Drug Targets.

[cit178] Chadwick S., Kriegel C., Amiji M. (2010). Adv. Drug Delivery Rev..

[cit179] Pokharkar V., Bhumkar D., Suresh K., Shinde Y., Gairola S., Jadhav S. S. (2011). J. Biomed. Nanotechnol..

[cit180] Pissuwan D., Nose K., Kurihara R., Kaneko K., Tahara Y., Kamiya N., Goto M., Katayama Y., Niidome T. (2011). Small.

[cit181] Kowalczyk D. W., Ertl H. C. J. (1999). Cell. Mol. Life Sci..

[cit182] Hasan U. A., Abai A. M., Harper D. R., Wren B. W., Morrow W. J. W. (1999). J. Immunol. Methods.

[cit183] Yang N. S. and Christou P., Particle Bombardment Technology for Gene Transfer, Oxford University Press, Oxford, 1994.

[cit184] O'Brien J. A., Lummis S. C. R. (2011). BMC Biotechnol..

[cit185] Donnelly J. J., Wahren B., Liu M. A. (2005). J. Immunol..

[cit186] DNA Vaccines: A New Era in Vaccinology, ed. M. A. Liu, M. R. Hillerman and R. Kurth, New York Academy of Sciences, New York, 1995.

[cit187] Gurunathan S., Klinman D. M., Seder R. A. (2000). Annu. Rev. Immunol..

[cit188] Yang J., Li Y., Jin S., Xu J., Wang P. C., Liang X.-J., Zhang X. (2015). Biomaterials Research.

[cit189] Sundaram P., Xiao W., Brandsma J. L. (1996). Nucleic Acids Res..

[cit190] Cui Z., Mumper R. J. (2003). Eur. J. Pharm. Biopharm..

[cit191] Zhang L., Widera G., Rabussay D. (2004). Bioelectrochemistry.

[cit192] Roy M. J., Wu M. S., Barr L. J., Fuller J. T., Tussey L. G., Speller S., Culp J., Burkholder J. K., Swain W. F., Dixon R. M., Widera G., Vessey R., King A., Ogg G., Gallimore A., Haynes J. R., Heydenburg Fuller D. (2000). Vaccine.

[cit193] Leutenegger C. M., Boretti F., Mislin C. N., Flynn J. N., Schroff M., Habel A., Junghans C., Koenig-Merediz S. A., Sigrist B., Aubert A., Pedersen N. C., Wittig B., Lutz H. (2000). J. Virol..

[cit194] Chen D., Payne L. G. (2002). Cell Res..

[cit195] Dean H. J., Fuller D., Osorio J. E. (2003). Comp. Immunol., Microbiol. Infect. Dis..

[cit196] Thomas M., Klibanov A. M. (2003). Proc. Natl. Acad. Sci. U. S. A..

[cit197] Salem A. K., Hung C. F., Kim T. W., Wu T. C., Searson P. C., Leong K. W. (2005). Nanotechnology.

[cit198] Xu L., Liu Y., Chen Z., Li W., Liu Y., Wang L., Liu Y., Wu X., Ji Y., Zhao Y., Ma L., Shao Y., Chen C. (2012). Nano Lett..

[cit199] Ojeda R., de Paz J. L., Barrientos A. G., Martín-Lomas M., Penadés S. (2007). Carbohydr. Res..

[cit200] Marradi M., Di Gianvincenzo P., Enríquez-Navas P. M., Martínez-Ávila O. M., Chiodo F., Yuste E., Angulo J., Penadés S. (2011). J. Mol. Biol..

[cit201] Brinãs R. P., Sundgren A., Sahoo P., Morey S., Rittenhouse-Olson K., Wilding G. E., Deng W., Barchi Jr J. J. (2012). Bioconjugate Chem..

[cit202] Parry A. L., Clemson N. A., Ellis J., Bernhard S. S. R., Davis B. G., Cameron N. R. (2013). J. Am. Chem. Soc..

[cit203] Mocan T., Matea C., Tabaran F., Iancu C., Orasan R., Mocan L. (2015). J. Cancer.

[cit204] Tavernaro I., Hartmann S., Sommer L., Hausmann H., Rohner C., Ruehl M., Hoffmann-Roeder A., Schlecht S. (2015). Org. Biomol. Chem..

[cit205] Cai H., Degliangeli F., Palitzsch B., Gerlitzki B., Kunz H., Schmitt E., Fiammengo R., Westerlind U. (2016). Bioorg. Med. Chem..

[cit206] Zhao Z., Wakita T., Yasui K. (2003). J. Virol..

[cit207] Dykman L. A., Bogatyrev V. A., Staroverov S. A., Pristensky D. V., Shchyogolev S. Y., Khlebtsov N. G. (2006). Proc. SPIE–Int. Soc. Opt. Eng..

[cit208] Dykman L. A., Staroverov S. A., Bogatyrev V. A. and Shchyogolev S. Y., Gold nanoparticles as an antigen carrier and an adjuvant, Nova Science Publishers, New York, 2010.

[cit209] Dykman L. A., Staroverov S. A., Bogatyrev V. A., Shchyogolev S. Y. (2010). Nanotechnology.

[cit210] Abraham G. E., Himmel P. B. (1997). J. Nutr.
Environ. Med..

[cit211] Abraham G. E. (2008). Original Internist.

[cit212] Tsai C. Y., Shiau A. L., Chen S. Y., Chen Y. H., Cheng P. C., Chang M. Y., Chen D. H., Chou C. H., Wang C. R., Wu C. L. (2007). Arthritis Rheum..

[cit213] Brown C. L., Bushell G., Whitehouse M. W., Agrawal D. S., Tupe S. G., Paknikar K. M., Tiekink E. R. T. (2007). Gold Bull..

[cit214] Graham G. (1993). Agents Actions Suppl..

[cit215] Merchant B. (1998). Biologicals.

[cit216] Eisler R. (2004). Biol. Trace Elem. Res..

[cit217] Vallhov H., Qin J., Johansson S. M., Ahlborg N., Muhammed M. A., Scheynius A., Gabrielsson S. (2006). Nano Lett..

[cit218] Kingston M., Pfau J. C., Gilmer J., Brey R. (2016). J. Immunotoxicol..

[cit219] Dobrovolskaia M. A., Germolec D. R., Weaver J. L. (2009). Nat. Nanotechnol..

[cit220] Zolnik B. S., González-Fernández A., Sadrieh N., Dobrovolskaia M. A. (2010). Endocrinology.

[cit221] Lin A. Y., Almeida J. P. M., Bear A., Liu N., Luo L., Foster A. E., Drezek R. A. (2013). PLoS One.

[cit222] Tao Y., Zhang Y., Ju E., Ren H., Ren J. (2015). Nanoscale.

[cit223] Zhou Q., Zhang Y., Du J., Li Y., Zhou Y., Fu Q., Zhang J., Wang X., Zhan L. (2016). ACS Nano.

[cit224] Zhang H., Gao X.-D. (2017). Mater. Sci. Eng., C.

[cit225] Wang Y., Wang Y., Kang N., Liu Y., Shan W., Bi S., Ren L., Zhuang G. (2016). Nanoscale Res. Lett..

[cit226] Klinman D. M., Sato T., Shimosato T. (2016). Wiley Interdiscip. Rev.: Nanomed. Nanobiotechnol..

[cit227] Zhang P., Chiu Y.-C., Tostanoski L. H., Jewell C. M. (2015). ACS Nano.

[cit228] Barhate G., Gautam M., Gairola S., Jadhav S., Pokharkar V. (2013). Int. J. Pharm..

[cit229] Barhate G., Gautam M., Gairola S., Jadhav S., Pokharkar V. (2014). J. Pharm. Sci..

[cit230] Joseph M. M., Aravind S. R., Varghese S., Mini S., Sreelekha T. T. (2013). Colloids Surf., B.

[cit231] Ye F., Vallhov H., Qin J., Daskalaki E., Sugunan A., Toprak M. S., Fornara A., Gabrielsson S., Scheynius A., Muhammed M. (2011). Int. J. Nanotechnol..

[cit232] Wang Y.-T., Lu X.-M., Zhu F., Zhao M. (2014). Bio. Med. Mater. Eng..

[cit233] Yavuz E., Sakalak H., Cavusoglu H., Uyar P., Yavuz M. S., Bagriacik E. U. (2016). Eur. J. Immunol..

[cit234] Jiao Q., Li L., Mu Q., Zhang Q. (2014). BioMed Res. Int..

[cit235] Zhang W., Wang L., Liu Y., Chen X., Liu Q., Jia J., Yang T., Qiu S., Ma G. (2014). Biomaterials.

[cit236] Comber J. D., Bamezai A. (2015). Journal of Nanomedicine & Biotherapeutic Discovery.

